# Association between trends in clinical variables and outcome in intensive care patients with faecal peritonitis: analysis of the GenOSept cohort

**DOI:** 10.1186/s13054-015-0931-8

**Published:** 2015-05-05

**Authors:** Ascanio Tridente, Geraldine M Clarke, Andrew Walden, Anthony C Gordon, Paula Hutton, Jean-Daniel Chiche, Paul AH Holloway, Gary H Mills, Julian Bion, Frank Stüber, Christopher Garrard, Charles Hinds, on behalf of the GenOSept Investigators

**Affiliations:** Intensive Care Unit, Whiston Hospital, Prescot, Warrington Road, Prescot, Merseyside L35 5DR UK; Department of Infection and Immunity, The Medical School, University of Sheffield, Beech Hill Rd, Sheffield, South Yorkshire, S10 2RX Sheffield, UK; The Wellcome Trust Centre for Human Genetics, University of Oxford, University Offices, Wellington Square, Oxford, OX1 2JD, Oxford UK; Intensive Care Unit, Royal Berkshire Hospital, Craven Road, RG1 5AN Reading, UK; Imperial College, SW7 2AZ London, UK; Intensive Care Unit, John Radcliffe Hospital, Headley Way, OX3 9DU Oxford, UK; Medical Intensive Care Unit, Hôpital Cochin, 27 rue du Faubourg Saint-Jacques, 75014 Paris, France; Intensive Care Unit, Sheffield Teaching Hospitals NHS Trust, Northern General Hospital, Herries Road, South Yorkshire, S5 7AU Sheffield, UK; Department of Anaesthesia and Critical Care, School of Clinical and Experimental Medicine, University of Birmingham, Office 1, Ground Floor East, old Queen Elizabeth Hospital, Edgbaston, Birmingham, B15 2TH UK; Department of Anaesthesiology and Pain Medicine, University Hospital Inselspital, Bern, and University of Bern, Bern, Switzerland; Barts and The School of Medicine and Dentistry, Queen Mary University of London, Turner Street, London, E1 2AD UK

## Abstract

**Introduction:**

Patients admitted to intensive care following surgery for faecal peritonitis present particular challenges in terms of clinical management and risk assessment. Collaborating surgical and intensive care teams need shared perspectives on prognosis. We aimed to determine the relationship between dynamic assessment of trends in selected variables and outcomes.

**Methods:**

We analysed trends in physiological and laboratory variables during the first week of intensive care unit (ICU) stay in 977 patients at 102 centres across 16 European countries. The primary outcome was 6-month mortality. Secondary endpoints were ICU, hospital and 28-day mortality. For each trend, Cox proportional hazards (PH) regression analyses, adjusted for age and sex, were performed for each endpoint.

**Results:**

Trends over the first 7 days of the ICU stay independently associated with 6-month mortality were worsening thrombocytopaenia (mortality: hazard ratio (HR) = 1.02; 95% confidence interval (CI), 1.01 to 1.03; P <0.001) and renal function (total daily urine output: HR =1.02; 95% CI, 1.01 to 1.03; P <0.001; Sequential Organ Failure Assessment (SOFA) renal subscore: HR = 0.87; 95% CI, 0.75 to 0.99; P = 0.047), maximum bilirubin level (HR = 0.99; 95% CI, 0.99 to 0.99; P = 0.02) and Glasgow Coma Scale (GCS) SOFA subscore (HR = 0.81; 95% CI, 0.68 to 0.98; P = 0.028). Changes in renal function (total daily urine output and renal component of the SOFA score), GCS component of the SOFA score, total SOFA score and worsening thrombocytopaenia were also independently associated with secondary outcomes (ICU, hospital and 28-day mortality). We detected the same pattern when we analysed trends on days 2, 3 and 5. Dynamic trends in all other measured laboratory and physiological variables, and in radiological findings, changes inrespiratory support, renal replacement therapy and inotrope and/or vasopressor requirements failed to be retained as independently associated with outcome in multivariate analysis.

**Conclusions:**

Only deterioration in renal function, thrombocytopaenia and SOFA score over the first 2, 3, 5 and 7 days of the ICU stay were consistently associated with mortality at all endpoints. These findings may help to inform clinical decision making in patients with this common cause of critical illness.

**Electronic supplementary material:**

The online version of this article (doi:10.1186/s13054-015-0931-8) contains supplementary material, which is available to authorized users.

## Introduction

Patients admitted to intensive care following surgery for faecal peritonitis (FP) present particular challenges in terms of clinical management and risk assessment. Collaborating surgical and intensive care teams need shared perspectives on trends in illness severity and likely outcomes. Dynamic assessment of trends and the response to treatment, including evaluation of changes in laboratory tests and dependence on organ support, may be more informative than isolated initial measurements when assessing the prognosis of individual patients. Several methods have been evaluated for dynamic assessment of critically ill patients [1-4], including those undergoing surgery, and some researchers have examined dynamic changes in patients with peritonitis [5-9]. The burden of data collection is considered by many to be a deterrent to routine use [10], however, and one study found that existing scoring systems were inadequate for this purpose [9]. These studies were relatively small, though; the largest reported data on 163 patients only.

Given the paucity of data on the association between trends in clinical variables during the early stages of intensive care unit (ICU) admission following surgical intervention for FP and outcomes, we used a large international database of patients with FP with the aim of analysing trends in all available clinical variables during the first week of ICU stay and their relationship to outcome.

Intraabdominal infections, and in particular FP, although affecting all age groups, are conditions which typically affect the elderly. The severity of sepsis and the likelihood of an adverse outcome are both reflected in persisting organ system failures and may be aggravated by limited physiologic reserve, the presence of comorbidities and impaired wound healing [11,12]. With an ageing population, the incidence of FP is likely to increase, adding pressure on already scarce health care resources [13,14]. In this setting, detecting trends in key laboratory and physiological variables and in the severity of organ dysfunction could prove useful in supporting decision making with regard to escalating, limiting or withdrawing treatment, and it might help reduce variability in critical care decision making [15].

The Genetics of Sepsis and Septic Shock in Europe (GenOSept) trial is a pan-European study funded by the Sixth Framework Programme of the European Union. It was conceived by the European Critical Care Research Network of the European Society for Intensive Care Medicine to investigate the potential impact of genetic variation on the host response and outcomes in sepsis [16]. To date, the GenOSept cohort includes the largest and diagnostically most homogeneous collection of clinical data on critically ill patients with FP. We recently reported outcome analyses of this cohort, based on data from day 1 of admission to the ICU [12].

## Material and methods

### Recruitment and ethical approval

Ethical approval was granted either nationally and/or locally (for individual centres—see Additional file 1). Written informed consent for inclusion in the GenOSept study was obtained from all patients or their legal representatives. The study was performed in accordance with the ethical standards laid down in the 1964 Declaration of Helsinki and its later amendments. Patients were recruited from 102 centres across 16 European countries (see Additional file 1 for contributors) between September 2005 and January 2011.

The definition of sepsis was based on the International Consensus Criteria as “the clinical syndrome defined by the presence of both infection and a systemic inflammatory response” ([17], p 532). Patients were followed for up to 6 months from enrolment or until death.

To be included, patients had to be adults (>18 years) admitted to a high-dependency unit or ICU with FP. FP was defined as inflammation of the serosal membrane that lines the abdominal cavity, secondary to contamination by faeces, as diagnosed at laparotomy.

Exclusion criteria were peritonitis due to gastric or upper gastrointestinal tract perforation (for example, gastric or duodenal ulcer perforation, terminal ileum perforation); patient or patient’s legal representative unwilling or unable to give consent; patient pregnant; advanced directive to withhold or withdraw life-sustaining treatment or admitted for palliative care only; patient already enrolled in an interventional research study of a novel and/or unlicensed therapy (patients enrolled in interventional studies examining the clinical application or therapeutic effects of widely accepted, “standard” treatments were not excluded); patient immunocompromised (known regular systemic corticosteroid therapy exceeding 7 mg/kg/day of hydrocortisone or equivalent within 3 months of admission and prior to acute episode, known regular therapy with other immunosuppressive agents (for example, azathioprine), known to be HIV-positive or to have AIDS as defined by the Centers for Disease Control and Prevention, neutrophil count <1,000/mm^3^ due to any cause, including metastatic disease and haematological malignancies or chemotherapy, but excluding severe sepsis; organ or bone marrow transplant recipient and receiving immunosuppressive therapy).

See Additional file 1 for information on database and quality assurance.

### Statistical analysis

Clinical data of patients with FP recruited to GenOSept were collected on days 1, 2, 3, 5 and 7 of the ICU stay, including all variables required to calculate admission Acute Physiology and Chronic Health Evaluation (APACHE) II and daily Sequential Organ Failure Assessment (SOFA) scores [18,19]. We used investigator-coded presence (or absence) of acute renal failure (ARF); the study was started before the international definitions of acute kidney injury (AKI) had been developed.

Data extracted from the electronic case report form for the purposes of this analysis pertained to the first week of ICU admission. The trends were calculated as linear change over the first week (obtained by subtracting the value on day 1 from the value on day 7) as a prospectively chosen summary measure for the primary analysis. The use of summary measures to combine data obtained at multiple time points for the same individual is a recommended statistical procedure. Use of such a measure allowed identification of a general trend over the first week of ICU stay while also avoiding common pitfalls in the analysis of serial measurements [20].

The primary study outcome was 6-month mortality. Secondary endpoints were ICU, hospital and 28-day mortality. We also evaluated shorter-term trends by subtracting the values on day 1 from the values on days 2, 3 and 5.

We analysed trends in all 35 variables where data were available for the first week of ICU stay. For each trend, Cox proportional hazards (PH) regression analyses adjusted for age and sex were performed for each endpoint. Trends found to be significant in these analyses, after Bonferroni correction for multiple testing (*P* value <0.00143 = 0.05/35 to take account of the 35 variables tested), were entered into a multivariate Cox PH model to identify the factors independently associated with mortality, adjusting for potential confounding factors. The full list of variables whose trends were tested is provided in Additional file 1.

Statistical analyses were performed using R version 2.11.1 (The R Project for Statistical Computing [21]) and STATA (StataCorp, College Station, TX, USA) [22] statistical software.

## Results

Between 29 September 2005 and 5 January 2011, 977 patients with a diagnosis of FP confirmed at laparotomy were recruited into the GenOSept cohort. Their median age was 69.2 (interquartile range (IQR), 58.3 to 77.1) years; 54.3% were men; and 98.6% of patients were Caucasian. The median ICU and hospital lengths of stay were 10 (IQR, 5 to 21) days and 28 (IQR, 15 to 51) days, respectively. The median APACHE II score was 16 (IQR, 12 to 21). Of these 977 patients, 187 (19.1%) had died at 28 days, 204 (20.9%) died during their ICU stay, 283 (28.7%) died in the hospital and 309 (31.6%) had died by the 6-month follow-up examination. Other admission characteristics of the GenOSept FP cohort were described in our previous publication [12].

Of 977 patients, 937 patients stayed in the ICU for at least 2 days; hence, they could be included in the analysis of dynamic trends. Of the remaining 40 (4.1%), 11 died on day 1, and 29 were discharged alive from the ICU on day 1 (of whom 21 were alive at 6 months and 8 were censored alive at hospital discharge). Also, 321 individuals did not have data for day 7, 237 did not have data at day 5 and 107 did not have data at day 3 (either because they had died or they had been discharged alive from ICU).

Table 1 describes trends in clinical variables between day 1 and day 7 of the ICU stay; for values related to days 2, 3 and 5, see Additional file 1: Table S1. During the observed period, the proportion of patients with ARF decreased from 29.1% (283 of 974) to 24.1% (159 of 659). The proportion of those receiving ventilatory support (as continuous positive airway pressure or invasive mechanical ventilation) fell from 76.3% (743 of 974) to 62.9% (413 of 657). The proportion of those receiving any inotropic and/or vasopressor therapy decreased from 72.6% (707 of 974) to 34.2% (226 of 660). The median (IQR) SOFA score decreased from 7 (5 to 10) to 5 (2 to 8), mainly due to a decrease in the cardiovascular and renal components.

**Table 1:** **Trends in variables during first 7 days of intensive care unit stay** ^**a**^

			**Day 1**			**Day 7**		
**Characteristics**			**N**	**n or median**	**% or IQR**	**N**	**n or median**	**% or IQR**
Organ failure and support								
	ARF		974	283	29.1%	659	159	24.1%
	RRT		974	115	11.8%	659	104	15.8%
	Ventilatory support		974	743	76.3%	657	413	62.9%
	Inotrope/vasopressor use^b^		974			660		
		None		267	27.4%		434	65.8%
		A		33	3.4%		36	5.5%
		B		169	17.4%		105	15.9%
		C		505	51.9%		85	12.9%
	SOFA		974	7	5 to 10	659	5	2 to 8
		GCS SOFA	974	0	0 to 1	674	0	0 to 1
		CVS SOFA	974	4	1 to 4	671	1	0 to 3
		Coagulation SOFA	974	0	0 to 1	674	0	0 to 1
		Respiratory SOFA	974	2	2 to 3	659	2	1 to 3
		Renal SOFA	974	1	0 to 2	674	0	0 to 1
		Bilirubin SOFA	974	0	0 to 1	674	0	0 to 0
Laboratory variables								
	Serum bicarbonate		893	21	18 to 24	580	26	23 to 29.1
	paO_2_ (kPa)		945	11.3	9.6 to 13.9	590	10.8	9.2 to 13.6
	paCO_2_ (kPa)		938	5.2	4.6 to 6	535	5.2	4.7 to 6
	Highest creatinine (μmol/L)		974	106.6	76 to 160	654	81.5	53.9 to 132
	Lowest creatinine (μmol/L)		974	100	70 to 144	642	81.5	54 to 130
	Highest WCC (10^−9^/L)		974	12	7.1 to 18	643	15.3	11 to 20.4
	Lowest WCC (10^−9^/L)		974	9.4	4.6 to 15	643	15	10.5 to 20
	Lowest platelets (10^−9^/L)		974	212.5	145 to 300	640	234.5	132 to 352.5
	Highest bilirubin (mmol/L)		974	13	8 to 22	650	10	5 to 19
	Highest urea (mmol/L)		908	11	6.4 to 17.1	607	11.6	7.2 to 20.8
Chest radiography findings								
	Localised infiltrates		973	115	11.8%	672	68	10.1%
	Lobar infiltrates		973	61	6.3%	672	32	4.8%
	Diffuse bilateral infiltrates		973	148	15.2%	672	109	16.2%
Physiological parameters								
	Highest temperature (°C)		974	37.7	37 to 38.3	656	37.4	37 to 38
	Lowest temperature (°C)		974	36.2	35.7 to 36.9	656	36.5	36 to 37
	Highest SBP (mmHg)		974	140	125 to 155	655	150	135 to 170
	Lowest SBP (mmHg)		972	90	80 to 100	655	108	95 to 120
	Highest MAP (mmHg)		972	91	82 to 103	649	98	89 to 110
	Lowest MAP (mmHg)		970	62	56 to 70	649	71	63 to 80
	Highest heart rate (bpm)		974	117	103 to 130	655	104	90 to 118
	Lowest heart rate (bpm)		971	85	73 to 99	655	80	70 to 90
	Respiratory rate (breaths/min)		966	18	14 to 24	638	20	16 to 27
	Urinary volume (ml/24 hr)		973	1,375	790 to 2,100	644	2,276.5	1,333.5 to 3,411.5
	paO_2_/FiO_2_ ratio (kPa)		942	27.8	19.6 to 37.7	573	30.7	23.1 to 39.2

^a^ARF, Acute renal failure; bpm, Beats per minute; CVS, Cardiovascular; GCS, Glasgow Coma Scale; IQR, Interquartile range; MAP, Mean arterial pressure; n, Absolute count; N, Number of non-missing observations; paCO_2_, Arterial partial pressure of carbon dioxide; paO_2_, Arterial partial pressure of oxygen; paO_2_/FiO_2_, Ratio of partial pressure of arterial oxygen to fraction of inspired oxygen; RRT, Renal replacement therapy; SBP, Systolic blood pressure; SOFA, Sequential Organ Failure Assessment; WCC, White cell count. ^b^Inotropic and vasopressor use is coded as follows: A = dopamine ≤5 μg/kg/min or dobutamine; B = dopamine >5 μg/kg/min or adrenaline/noradrenaline ≤0.1 μg/kg/min; C = dopamine >15 μg/kg/min or adrenaline/noradrenaline >0.1 μg/kg/min.

Table 2 shows the estimated hazard ratios (HRs) for the primary and secondary endpoints for trends in variables that were significant after adjusting for multiple testing in individual variable analyses and after inclusion in multivariate analyses.

**Table 2:** **Factors independently associated with 6**-**month**, **intensive care unit**, **hospital and 28**-**day mortality**, **after adjustment for age and sex** ^**a**^

**Variable**		**HR**	**95% ** **CI**	***P***-**value**
6-month mortality				
	Deterioration of thrombocytopaenia (10 × 10^−9^/L platelets)	1.02	1.01 to 1.03	<0.001
	Decrease in daily urinary volume (100 ml)	1.02	1.01 to 1.03	<0.001
	Decrease in highest recorded bilirubin (mmol/L)	0.99	0.99 to 0.99	0.020
	Improvement in GCS SOFA score (1 point)	0.81	0.68 to 0.98	0.028
	Improvement in renal SOFA score (1 point)	0.87	0.75 to 0.99	0.047
ICU mortality				
	Decrease in daily urinary volume (100 ml)	1.02	1.01 to 1.03	0.005
	Improvement in total SOFA score (1 point)	0.91	0.85 to 0.98	0.009
Hospital mortality				
	Deterioration of thrombocytopaenia (10 × 10^−9^/L platelets)	1.02	1.01 to 1.03	0.001
	Decrease in daily urinary volume (100 ml)	1.02	1.01 to 1.03	<0.001
	Improvement in GCS SOFA score (1 point)	0.8	0.68 to 0.95	0.011
	Improvement in renal SOFA score (1 point)	0.87	0.77 to 0.99	0.043
28-day mortality				
	Decrease in daily urinary volume (100 ml)	1.02	1.01 to 1.03	0.001
	Improvement in GCS SOFA score (1 point)	0.75	0.61 to 0.93	0.010
	Improvement in renal SOFA score (1 point)	0.79	0.66 to 0.95	0.013

^a^CI, Confidence interval; GCS, Glasgow Coma Scale; HR, Hazard ratio; ICU, Intensive care unit; SOFA, Sequential Organ Failure Assessment.

The trends in variables over the first 7 days of the ICU stay that remained significantly and independently associated with 6-month outcome were worsening thrombocytopaenia and renal function (total daily urine output and renal component of the SOFA score), highest recorded level of bilirubin and GCS component of the SOFA score.

Changes in renal function (total daily urine output and renal component of the SOFA score), GCS component of the SOFA score, total SOFA and worsening thrombocytopaenia were also independently associated with secondary outcomes.

Dynamic trends in all other measured laboratory and physiological variables and in radiological findings failed to be independently associated with outcome on multivariate analyses. Furthermore, changes in respiratory support, renal replacement therapy and inotrope and/or vasopressor requirement appeared not to be independently associated with any of the primary or secondary outcomes.

**Figure 1:**
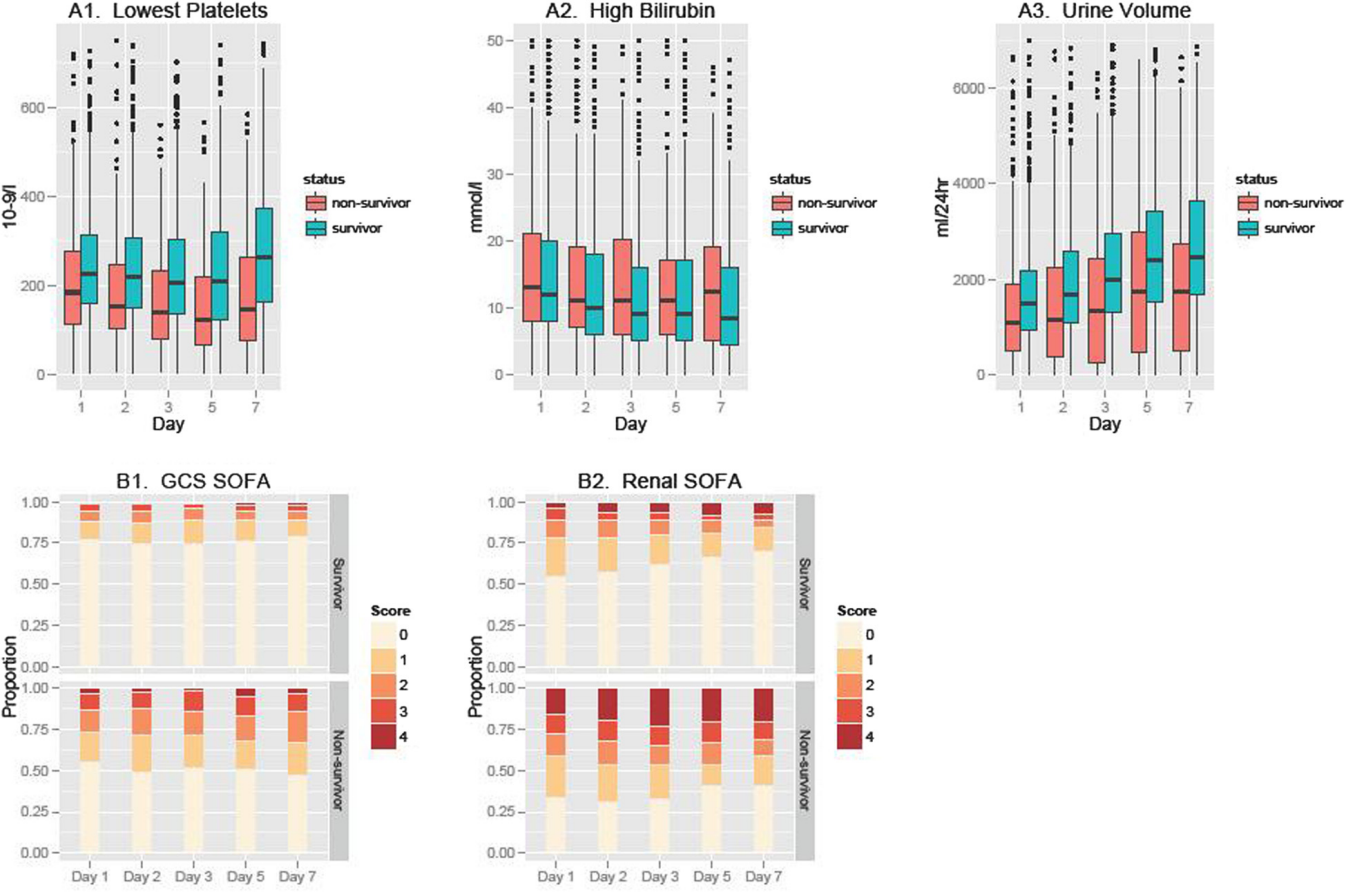
Trends in variables independently associated with 6-month survival (primary outcome). **(A1)** to **(A3)** Daily lowest platelet count, 24-hour urinary volume and highest recorded bilirubin concentration. The boxes indicate median and interquartile range (IQR), the whiskers extend to include 1.5× IQR and the dots include outliers outside this range. **(B1)** and **(B2)** Daily Glasgow Coma Scale (GCS) score and renal components of the Sequential Organ Failure Assessment (SOFA) score. Proportions of different values of the renal and GCS components of the SOFA are indicated for survivors and non-survivors.

Figure 1 displays trends over the first week of ICU admission in the five variables independently associated with 6-month outcome (thrombocytopaenia, daily urinary volume, renal and GCS components of the SOFA score, serum bilirubin concentration). Survivors and non-survivors displayed differences in both absolute values and trends in these variables during their ICU stay. The platelet count remained consistently lower throughout the observation period and decreased more markedly in non-survivors compared with survivors. The daily total urine output was consistently greater and increased more markedly in survivors compared with non-survivors throughout the 7 days (Table 3). The highest measured bilirubin level showed an improvement during the 7-day observation period in survivors, but not in non-survivors. The GCS component of the SOFA score for non-survivors tended to be initially worse and to deteriorate further during the observation period, and the renal component of the SOFA score tended to be consistently worse in non-survivors.

**Table 3:** **Trends during first 7 days of the intensive care unit stay** (**in survivors and non**-**survivors at 6 months**) **for variables independently associated with outcomes in multivariate analyses** ^**a**^

		**Day 1**			**Day 7**		
**Characteristics**		**N**	**Median**	**IQR**	**N**	**Median**	**IQR**
Lowest platelets (*10^9^/L)							
	Survivors	667	226	159 to 316	426	267	164 to 388
	Non-survivors	307	185	113 to 280	214	146	78 to 274
Urinary volume (ml/24 hours)							
	Survivors	666	1,499	916 to 2,170	429	2460	1,680 to 3,633
	Non-survivors	307	1,080	475 to 1,915	215	1750	471 to 2,774
Highest bilirubin (mmol/L)							
	Survivors	667	13	8 to 21	433	9	5 to 17
	Non-survivors	307	14	8 to 25	217	14	6 to 25
GCS SOFA score							
	Survivors	667	0	0 to 0	453	0	0 to 0
	Non-survivors	307	0	0 to 2	221	1	0 to 2
Renal SOFA score							
	Survivors	667	0	0 to 1	453	0	0 to 1
	Non-survivors	307	1	0 to 3	221	1	0 to 3

^a^GCS, Glasgow Coma Scale; IQR, Interquartile range; n, Absolute count; N, Number of non-missing observations; SOFA, Sequential Organ Failure Assessment.

The analyses of trends over the first 2, 3 and 5 days yielded similar results. The trends over the first 48 hours of the ICU stay independently associated with outcomes were deteriorating thrombocytopaenia and renal function (urinary output and renal SOFA score) and total SOFA score (Additional file 1: Table S2). The trends over the first 3 days in the ICU independently associated with outcomes were thrombocytopaenia, renal function (urinary output and renal SOFA score), total SOFA score and, for 28-day mortality only, GCS SOFA score, mean arterial pressure and ratio of partial pressure of arterial oxygen to fraction of inspired oxygen (Additional file 1: Table S3). The trends over the first 5 days in the ICU independently associated with outcomes were thrombocytopaenia, renal function (urinary output, renal SOFA score and highest recorded serum urea) and total SOFA score (Additional file 1: Table S4).

## Discussion

This large, prospectively gathered, diagnostically homogeneous and rigorously quality-assured European cohort provides a unique opportunity to examine associations between trends in clinical variables and short- and long-term outcomes of postoperative patients with FP admitted to ICUs.

In our study, the trends most strongly associated with mortality in all the analyses were worsening thrombocytopaenia and renal dysfunction, SOFA score and, in particular, the GCS and renal components of the SOFA score.

In two studies published as conference abstracts, researchers have specifically evaluated outcomes of critically ill patients with FP, with different mortality rates reported [23]. Sayer *et al*. reported in-hospital mortality rates of 21.6% and 38.1% for the malignancy and non-malignancy subgroups, respectively [23,24], whereas Pawa *et al*. reported 30-day mortality rates of 46% for patients aged <75 years and 78% for patients aged >75 years, suggesting that different local practices of critical care admission, periods considered, evolution in concurrent treatments and underlying characteristics of the populations (other than admission diagnosis) may have influenced their results. None of the studies involved microbiological isolates or the relationship between trends in clinical variables and outcomes. Pawa and coworkers reported age as the strongest outcome predictor, whereas Sayer and colleagues suggested that hypoalbuminaemia and the presence of malignancy influence mortality [24]. In other studies, researchers have evaluated heterogeneous populations of patients with peritonitis of multiple aetiologies, with several different predictors identified, depending on the patient case mix [9,25,26].

### Worsening thrombocytopaenia

In our cohort, worsening thrombocytopaenia over the first 7 days of the ICU stay was found to be independently associated with the primary outcome (6-month mortality) and two of the secondary outcomes (hospital and 28-day mortality). Thrombocytopaenia is a common finding following operative intervention for intraabdominal sepsis, and a falling platelet count has been reported to be useful for distinguishing infected from non-infectious peritonitis [27]. Thrombocytopaenia is also a marker of disease severity, coadministration of blood products and development of consumption coagulopathy. A strong link between poor outcome of critically ill patients and both low absolute platelet counts and the development and/or worsening of thrombocytopaenia have also previously been reported in various patient populations [28-32]. In the study by Vanderschueren and colleagues, who studied an unselected population of intensive care patients, the development of thrombocytopaenia and a reduction from baseline of 50% or more in platelet count had more explanatory power for ICU mortality than admission variables [28]. Williamson *et al*. examined the effects of prevalent and incident thrombocytopaenia in an unselected population of over 20,000 critically ill patients and demonstrated an independent association of low platelet counts with mortality. This association was stronger for specific admission diagnoses, in particular the cancer, respiratory, digestive, genitourinary and infectious categories [29]. In the study by Strauss *et al*., a decrease in platelet count ≥30% was significantly linked to higher mortality in 145 unselected critically ill patients [30]. Sharma *et al*., in a prospective observational study, evaluated the incidence of various degrees of severity of thrombocytopaenia in 69 patients with septic shock and concluded that thrombocytopaenia is associated with worse clinical outcomes in their unselected population of critically ill patients [31]. Crowther *et al*. also found that the development of thrombocytopaenia was strongly associated with mortality in 261 unselected critical care patients [32].

### Deteriorating renal function

In the GenOSept FP cohort, worsening renal function was consistently found to be associated with higher mortality at all time points. Although there is still some debate as to whether the excess mortality observed with renal dysfunction simply reflects the severity of the underlying illness or whether renal dysfunction independently contributes to poorer survival, the retention of this relationship on multivariate analysis suggests that the development of AKI directly contributes to worse outcomes. Impaired renal function has also been linked to impaired immune function [33-36]. In the study by Barrantes *et al*. of 496 critically ill patients, the mortality rate of patients with AKI was significantly higher than those without [33]. Ostermann *et al*. examined the effect of AKI on over 22,000 adult general ICU patients and found that the AKI classification correlated with outcome [34]. In a study conducted in an unselected ICU population of almost 42,000 patients, the same authors examined the criteria for acute renal injury, acute renal failure syndrome and severe acute renal failure syndrome and found that worsening degrees of renal impairment correlated with mortality [35]. Mehta *et al*. studied 611 unselected ICU patients and highlighted the higher incidence of sepsis amongst those with AKI [36].

### Deteriorating Sequential Organ Failure Assessment score

Although the SOFA score was originally conceived as a tool for describing the evolution of dysfunction in various organs rather than to predict outcome, we found that trends in the global SOFA score and in specific components (renal and GCS) were closely associated with mortality [19]. Our findings are comparable with those of a recently reported prospective observational cohort study in which the researchers investigated the systems that most contribute to the development of multiple organ system failure (MOSF). That study of 102 patients with abdominal sepsis highlighted the importance of trends in the SOFA score, demonstrating how the value on day 4 (as opposed to admission SOFA) had a high precision in predicting 28-day mortality, with MOSF being contributed to mainly by renal, central nervous system and respiratory system dysfunction [5]. In another study, which included 62 critically ill patients with postoperative peritonitis, investigators demonstrated the importance of trends in SOFA scores calculated serially over a 5-day postoperative period to distinguish between patients with or without persistent postoperative intraabdominal sepsis [6]. In a prospective observational study of 56 patients with secondary peritonitis, researchers measured several inflammatory parameters and multiple severity scoring systems preoperatively and over a 5-day postoperative period in a serial fashion. That study showed that combining the SOFA scores with measurement of serum neopterin concentration (a specific cellular immune system activation marker) and tumour necrosis factor receptor 2 levels yielded the highest predictive sensitivities and specificities for pre- and postoperative outcomes [7]. In a study of 163 consecutive ICU patients with secondary peritonitis, hospital mortality was accurately predicted by the postoperative SOFA score [8]. In a large trial comparing on-demand versus planned relaparotomy for severe peritonitis, the SOFA score showed good discriminatory power to predict hospital mortality, although it was unable to predict the need for relaparotomy [9,37]. In a retrospective cohort study by Sumi *et al*., both the SOFA score and Physiological and Operative Severity Score for the enUmeration of Mortality and Morbidity were able to stratify patients’ risk for undergoing surgical intervention for colorectal perforation [38].

Mulier *et al*. also previously reported the influence of coma on mortality in a study of generalized postoperative patients with peritonitis independently of age and source control [39]. Similarly, Matsumura *et al*. studied 218 general medical and surgical ICU patients and showed that serum procalcitonin levels and SOFA score at ICU discharge could predict post-ICU mortality and survival time [40]. Jones *et al*. studied 248 emergency department patients with severe sepsis and evidence of hypoperfusion at presentation, and they concluded that the SOFA score had prognostic value for in-hospital survival [41].

### Hyperbilirubinaemia

Postoperative hyperbilirubinaemia has been linked to persistent postsurgical infection and a poor prognosis [42]. In a study of patients with peritonitis, hyperbilirubinaemia, together with age and organ and/or system failures, was found to be amongst a number of mortality predictors in univariate analysis [43].

Although the HRs presented above in Table 2 are small, the larger the change in the underlying variable, the larger the effect (proportionally) on mortality. For example, a reduction in platelet count of 50 (*10^9^/L) is associated with a HR for 6-month mortality of 1.10 (95% confidence interval (CI), 1.05 to 1.16; *P* <0.001), indicating a 10% increase in risk of death at 6 months. Similarly, a reduction in 24 hour urine output of just 500 ml is associated with a HR for 6 month mortality of 1.09 (95% CI, 1.04 to 1.14; *P* <0.001), which translates into a 9% increase in risk of death. Such trends are therefore potentially relevant to the practicing clinician and will contribute to bedside assessment of severity of illness in patients with FP.

### Limitations

The main limitation of our study is the potential for unmeasured factors to confound the associations detected. Subsequent prospective studies specifically aimed at confirming the potential predictive accuracy of the trends identified here are required to further assess their value in clinical practice. The overwhelming majority of patients enrolled in this study were Caucasian; therefore, caution should be exercised when extrapolating these findings to different ethnic populations. Our findings are applicable to patients with FP who stayed in the ICU for at least 2 days, although only 40 (4.1%) of the 977 patients with FP in this cohort had a shorter stay; hence, we consider it unlikely that this generated significant bias.

## Conclusions

In this large and homogeneous cohort of critically ill patients with FP admitted to European ICUs, we have shown that changes in routinely measured clinical, physiological and laboratory parameters, readily available at the bedside, are associated with outcome. Only deterioration in renal function, thrombocytopaenia and SOFA score over the first 2, 3, 5 and 7 days were consistently associated with mortality. Other laboratory variables, radiological findings, physiological parameters or even changes in respiratory support, renal replacement therapy and inotrope and/or vasopressor requirements, as analysed here over multiple time intervals, appeared not to be independently and consistently associated with any of the primary or secondary outcomes.

Patients displaying deteriorating trends in these key clinical variables may be prospectively identified as at higher mortality risk. External validation of the predictive power of the trends identified here would clarify to what extent detection of such trends could be relied upon to inform clinical decision making.

## Key messages

- In critically ill patients with faecal peritonitis, only deterioration in renal function, thrombocytopaenia and SOFA score over the first 2, 3, 5 and 7 days were consistently associated with mortality.
- Other laboratory variables, radiological findings, physiological parameters or changes in respiratory support, renal replacement therapy and inotrope and/or vasopressor requirements were not independently associated with any of the primary or secondary outcomes.

## Additional file

Additional file 1: **Supplementary material and information.**

## Abbreviations

AKI: Acute kidney injury
APACHE: Acute Physiology and Chronic Health Evaluation
ARF: Acute renal failure
bpm: Beats per minute
CI: Confidence interval
CVS: Cardiovascular
FP: Faecal peritonitis
GCS: Glasgow Coma Scale
GenOSept: Genetics of Sepsis and Septic Shock in Europe
HR: Hazard ratio
ICU: Intensive care unit
IQR: Interquartile range
MAP: Mean arterial pressure
MOSF: Multiple organ system failure
N: Number of non-missing observations
paO_2_/FiO_2_: Ratio of partial pressure of arterial oxygen to fraction of inspired oxygen
paCO_2_: Arterial partial pressure of carbon dioxide
paO_2_: Arterial partial pressure of oxygen
RRT: Renal replacement therapy
SBP: Systolic blood pressure
SOFA: Sequential Organ Failure Assessment
WCC: White cell count
