# Supplementary material for: Association between trends in clinical variables and outcome in intensive care patients with faecal peritonitis: analysis of the GenOSept cohort
Source: Crit Care. 2015 May 5;19(1):210. doi: 10.1186/s13054-015-0931-8 (PMC4432819; doi:10.1186/s13054-015-0931-8)
Supplement: Supplementary file 1 — Supplementary material and information. [file 13054_2015_931_MOESM1_ESM.doc]

**GenOSept Faecal Peritonitis supplement**

Table S1. Trends in variables (days 2, 3 and 5 ICU stay); n, absolute count; IQR, interquartile range; N, number of non-missing observations; SOFA, Sequential Organ Failure Assessment; GCS, Glasgow Coma Scale; ARF, Acute Renal Failure; RRT, Renal Replacement Therapy; CVS, cardiovascular; paO_2_, arterial partial pressure of oxygen; paCO_2_, arterial partial pressure of carbon dioxide; WCC, White Cell Count; SBP, Systolic Blood Pressure; MAP, Mean Arterial Pressure; bpm, beats per minute;

^a^Median and ^b^IQR are shown instead of absolute count (n) and percentage (%).

| **Day** | **Day 2** | | | **Day 3** | | | **Day 5** |  |  |
| --- | --- | --- | --- | --- | --- | --- | --- | --- | --- |
| **Characteristics** | **N** | **n or Median^a^** | **% or IQR^b^** | **N** | **n or Median^a^** | **% or IQR^b^** | **N** | **n or Median^a^** | **% or IQR^b^** |
| **Organ failure and support** |  |  |  |  |  |  |  |  |  |
| ARF | 939 | 274 | 29.2 | 873 | 251 | 28.8 | 742 | 203 | 27.4 |
| RRT | 939 | 151 | 16.1 | 873 | 158 | 18.1 | 743 | 132 | 17.8 |
| Ventilatory support | 938 | 627 | 66.8 | 872 | 569 | 65.3 | 742 | 490 | 66 |
| Inotropes/vasopressors use* | 938 |  |  | 873 |  |  | 742 |  |  |
| None |  | 276 | 29.4 |  | 345 | 39.5 |  | 421 | 56.7 |
| A |  | 31 | 3.3 |  | 50 | 5.7 |  | 47 | 6.3 |
| B |  | 209 | 22.3 |  | 192 | 22 |  | 119 | 16 |
| C |  | 422 | 45 |  | 286 | 32.8 |  | 155 | 20.9 |
| SOFA | 939 | 7^a^ | 4-10^b^ | 873 | 7^a^ | 3-9^b^ | 743 | 6^a^ | 3-9^b^ |
| GCS SOFA | 940 | 0^a^ | 0-1^b^ | 877 | 0^a^ | 0-1^b^ | 755 | 0^a^ | 0-1^b^ |
| CVS SOFA | 939 | 3^a^ | 1-4^b^ | 877 | 3^a^ | 0-4^b^ | 752 | 1^a^ | 0-3^b^ |
| Coagulation SOFA | 940 | 0^a^ | 0-1^b^ | 877 | 0^a^ | 0-1^b^ | 755 | 0^a^ | 0-1^b^ |
| Respiratory SOFA | 939 | 2^a^ | 1-3^b^ | 873 | 2^a^ | 1-3^b^ | 743 | 2^a^ | 1-3^b^ |
| Renal SOFA | 940 | 1^a^ | 0-2^b^ | 877 | 0^a^ | 0-2^b^ | 755 | 0^a^ | 0-2^b^ |
| Bilirubin SOFA | 940 | 0^a^ | 0-1^b^ | 877 | 0^a^ | 0-0^b^ | 755 | 0^a^ | 0-0^b^ |
| **Laboratory parameters** |  |  |  |  |  |  |  |  |  |
| Serum bicarbonate | 845 | 22.4^a^ | 20-25,6^b^ | 782 | 24^a^ | 21-27^b^ | 657 | 25.2^a^ | 22.9-29^b^ |
| paO_2_ (kPa) | 883 | 10.9^a^ | 9.6-13.2^b^ | 807 | 11^a^ | 9.2-13.3^b^ | 679 | 10.8^a^ | 9.1-13.2^b^ |
| paCO_2_ (kPa) | 819 | 5.3^a^ | 4.7-6.1^b^ | 749 | 5.3^a^ | 4.7-6.1^b^ | 623 | 5.3^a^ | 4.7-6^b^ |
| Highest creatinine (µmol/l) | 938 | 105.3^a^ | 75-158^b^ | 870 | 96.4^a^ | 68-150^b^ | 740 | 87^a^ | 59.3-141^b^ |
| Lowest creatinine (µmol/l) | 935 | 100^a^ | 70-150^b^ | 869 | 94^a^ | 66-146^b^ | 732 | 85.4^a^ | 58.3-136^b^ |
| Highest WCC (10^-9^/l) | 937 | 13.2^a^ | 9.3-19^b^ | 870 | 14.3^a^ | 9.8-18.9^b^ | 725 | 13.5^a^ | 10.2-18.9^b^ |
| Lowest WCC (10^-9^/l) | 937 | 12.4^a^ | 8.4-18^b^ | 869 | 13.2^a^ | 9.4-18.4^b^ | 725 | 13^a^ | 9.8-18.1^b^ |
| Lowest platelets (10^-9^/l) | 937 | 204^a^ | 130-294^b^ | 868 | 189^a^ | 115-286^b^ | 726 | 180.5^a^ | 102-285^b^ |
| Highest bilirubin (mmol/l) | 938 | 11^a^ | 6.8-20^b^ | 871 | 10^a^ | 6-18^b^ | 739 | 10^a^ | 5-19^b^ |
| Highest urea (mmol/l) | 874 | 10.7^a^ | 7-18^b^ | 815 | 10.6^a^ | 6.7-18^b^ | 687 | 11.4^a^ | 6.6-20.1^b^ |
| **Chest radiography findings** | |  |  |  |  |  |  |  |  |
| Localised infiltrates | 939 | 76 | 8.1 | 876 | 78 | 8.9 | 752 | 77 | 10.2 |
| Lobar infiltrates | 939 | 37 | 3.9 | 876 | 28 | 3.2 | 752 | 43 | 5.7 |
| Diffuse bilateral infiltrates | 939 | 109 | 11.6 | 876 | 122 | 13.9 | 752 | 126 | 16.8 |
| **Physiological parameters** |  |  |  |  |  |  |  |  |  |
| Highest temperature (°C) | 936 | 37.5^a^ | 37-38^b^ | 870 | 37.3^a^ | 36.9-38^b^ | 740 | 37.4^a^ | 36.9-38^b^ |
| Lowest temperature (°C) | 936 | 36.4^a^ | 36-37^b^ | 870 | 36.4^a^ | 36-36.9^b^ | 740 | 36.4^a^ | 36-37^b^ |
| Highest SBP (mmHg) | 938 | 142^a^ | 130-160^b^ | 872 | 150^a^ | 130.5-168^b^ | 740 | 151^a^ | 135-170^b^ |
| Lowest SBP (mmHg) | 936 | 100^a^ | 90-110^b^ | 872 | 105^a^ | 93-118^b^ | 738 | 110^a^ | 96-120^b^ |
| Highest MAP (mmHg) | 929 | 95^a^ | 84-105^b^ | 860 | 98^a^ | 88-110^b^ | 733 | 100^a^ | 88-112^b^ |
| Lowest MAP (mmHg) | 927 | 66^a^ | 60-74^b^ | 859 | 70^a^ | 61-78^b^ | 730 | 70^a^ | 64-80^b^ |
| Highest Heart Rate (bpm) | 938 | 110^a^ | 98-125^b^ | 872 | 106^a^ | 94-120^b^ | 740 | 103^a^ | 90-119^b^ |
| Lowest Heart Rate (bpm) | 935 | 83^a^ | 72-95^b^ | 871 | 80^a^ | 70-90^b^ | 737 | 80^a^ | 70-90^b^ |
| Respiratory rate (breath/minute) | 929 | 18^a^ | 15-24^b^ | 861 | 20^a^ | 15-24^b^ | 729 | 20^a^ | 16-26^b^ |
| Urine volume (ml/24 hours) | 931 | 1580^a^ | 926-2460^b^ | 868 | 1869^a^ | 1012.5-2882.5^b^ | 733 | 2200^a^ | 1280-3375^b^ |
| P:F ratio (kPa) | 880 | 29.3^a^ | 21-39.1^b^ | 803 | 30.3^a^ | 21.4-38.6^b^ | 666 | 30.7^a^ | 22.3-39.1^b^ |

* Inotropic and vasopressors use was coded as follows: A = Dopamine ≤ 5 μg/kg/min or Dobutamine, B = Dopamine > 5 μg/kg/min or adrenaline/noradrenaline ≤0.1 μg/kg/min, C = Dopamine > 15 μg/kg/min or adrenaline/noradrenaline > 0.1 μg/kg/min

Table S2. Analyses conducted on trends over the first 2 days ICU stay showing factors independently associated with 6 month, ICU, hospital and 28 day mortality, after adjustment for age and gender. SOFA; HR, Hazard Ratio; CI, confidence interval

| **Variable** | **Unit** | **HR** | **95% CI** | **p value** |
| --- | --- | --- | --- | --- |
| **6 month mortality** |  |  |  |  |
| Deterioration of thrombocytopaenia | 10 x 10^-9^/l platelets | 1.025 | 1.01-1.04 | 0.004 |
| Improvement in total SOFA | 1 point | 0.93 | 0.87-0.99 | 0.02 |
| Decrease in daily urinary volume | 100 ml | 1.01 | 1-1.02 | 0.025 |
| **ICU mortality** |  |  |  |  |
| Improvement in renal SOFA | 1 point | 0.84 | 0.71-0.99 | 0.05 |
| **Hospital mortality** |  |  |  |  |
| Improvement in total SOFA | 1 point | 0.93 | 0.87-0.99 | 0.03 |
| Improvement in renal SOFA | 1 point | 0.86 | 0.75-0.99 | 0.04 |
| **28 day mortality** |  |  |  |  |
| Improvement in total SOFA | 1 point | 0.87 | 0.8-0.94 | <0.001 |

Table S3. Analyses conducted on trends over the first 3 days ICU stay showing factors independently associated with 6 month, ICU, hospital and 28 day mortality, after adjustment for age and gender. SOFA, Sequential Organ Failure Assessment, GCS, Glasgow Coma Score; MAP, Mean Arterial Pressure; P:F, ratio of partial pressure arterial oxygen and fraction of inspired oxygen; HR, Hazard Ratio; CI, confidence interval

| **Variable** | **Unit** | **HR** | **95% CI** | **p value** |
| --- | --- | --- | --- | --- |
| **6 month mortality** |  |  |  |  |
| Deterioration of thrombocytopaenia | 10 x 10^-9^/l platelets | 1.03 | 1.02-1.05 | <0.001 |
| Improvement in renal SOFA | 1 point | 0.78 | 0.69-0.89 | <0.001 |
| Decrease in daily urinary volume | 100 ml | 1.01 | 1-1.02 | 0.040 |
| **ICU mortality** |  |  |  |  |
| Improvement in renal SOFA | 1 point | 0.84 | 0.73-0.97 | 0.016 |
| Improvement in total SOFA | 1 point | 0.92 | 0.87-0.97 | 0.004 |
| **Hospital mortality** |  |  |  |  |
| Deterioration of thrombocytopaenia | 10 x 10^-9^/l platelets | 1.02 | 1.01-1.04 | 0.014 |
| Improvement in total SOFA | 1 point | 0.94 | 0.89-0.99 | 0.025 |
| Improvement in renal SOFA | 1 point | 0.81 | 0.71-0.92 | 0.001 |
| **28 day mortality** |  |  |  |  |
| Worsening in P:F ratio | 1 kPa | 1.03 | 1.02-1.04 | <0.001 |
| Improvement in GCS SOFA | 1 point | 0.78 | 0.62-0.98 | 0.033 |
| Improvement in renal SOFA | 1 point | 0.74 | 0.61-0.89 | 0.002 |
| Deterioration in lowest MAP | 1 mmHg | 1.02 | 1-1.03 | 0.024 |

Table S4. Analyses conducted on trends over the first 5 days ICU stay showing factors independently associated with 6 month, ICU, hospital and 28 day mortality, after adjustment for age and gender. SOFA, Sequential Organ Failure Assessment; HR, Hazard Ratio; CI, confidence interval

| **Variable** | **Unit** | **HR** | **95% CI** | **p value** |
| --- | --- | --- | --- | --- |
| **6 month mortality** |  |  |  |  |
| Deterioration of thrombocytopaenia | 10 x 10^-9^/l platelets | 1.02 | 1.01-1.03 | 0.003 |
| Improvement in renal SOFA | 1 point | 0.74 | 0.56-0.99 | 0.040 |
| **ICU mortality** |  |  |  |  |
| Improvement in total SOFA | 1 point | 0.92 | 0.88-0.97 | 0.001 |
| **Hospital mortality** |  |  |  |  |
| Deterioration of thrombocytopaenia | 10 x 10^-9^/l platelets | 1.02 | 1-1.03 | 0.025 |
| Decrease in daily urinary volume | 100 ml | 1.01 | 1-1.02 | 0.010 |
| Improvement in total SOFA | 1 point | 0.89 | 0.81-0.99 | 0.030 |
| **28 day mortality** |  |  |  |  |
| Improvement in renal SOFA | 1 point | 0.81 | 0.69-0.96 | 0.015 |
| Improvement in highest recorded urea | mmol/l | 0.99 | 0.98-0.99 | <0.001 |

**List of variables available for trends testing**

**Variables related to organ failure and support**

Presence of acute renal failure (ARF)

Need for renal replacement therapy (RRT)

Need for ventilatory support

Total SOFA (Sequential Organ Failure Assessment) score

GCS (Glasgow Coma Scale) SOFA score

CVS (Cardiovascular) SOFA score

Coagulation SOFA score

Respiratory SOFA score

Renal SOFA score

Bilirubin SOFA score

**Laboratory parameters**

Serum bicarbonate

Arterial partial pressure of O_2_

Arterial partial pressure of CO_2_

Highest recorded serum creatinine

Lowest recorded serum creatinine

Highest recorded WCC (White Cell Count)

Lowest recorded WCC

Lowest recorded platelets

Highest recorded serum bilirubin

Highest recorded serum urea

**Radiological changes**

Localised infiltrates on chest radiography

Lobar infiltrates on chest radiography

Diffuse bilateral infiltrates on chest radiography

**Physiological parameters**

Highest recorded temperature

Lowest recorded temperature

Highest recorded SBP (systolic blood pressure)

Lowest recorded SBP

Highest recorded MAP (mean arterial pressure)

Lowest recorded MAP

Highest recorded Heart Rate

Lowest recorded Heart Rate

Respiratory rate

Urine volume

P:F ratio

Presence of hypertension

**List of Study Personnel Responsible for Database Development and Quality Control**.

C.H. – Charles Hinds, Professor of Intensive Care Medicine, William Harvey Research Institute, Barts and the London Queen Mary School of Medicine

C.G. – Dr Chris Garrard, Consultant in Intensive Care Medicine, John Radcliffe Hospital, Oxford.

A.G. – Dr Anthony Gordon, Clinical Senior Lecturer and Consultant in Critical Care Medicine, Imperial College London.

P.H. – Ms Paula Hutton, chief research nurse, John Radcliffe Hospital, Oxford

A.W. – Dr Andrew Walden, Consultant in Acute and Intensive Care Medicine Royal Berkshire Hospital, Intensive Care Unit, Berkshire, UK

J-D.C. – Dr Jean-Daniel Chiche, Réanimation Médicale, Hȏpital Cochin, Paris, France

**Funding**

GenOSept (Genetics Of Sepsis and Septic Shock in Europe) is a pan-European part-FP6-funded study conceived by the European Critical Care Research Network of the European Society for Intensive Care Medicine to investigate the potential impact of genetic variation on the host response and outcomes in sepsis (https://www.genosept.eu/).

CIBERES is a Spanish research network which was used to identify investigators and contributed to funding through supporting logistics. A grant in partial support of FP6 projects was provided by the Spanish minister of Health.

Database and quality assurance

The case report form (CRF) was developed and tested by CH, CG, AG, JDC and Dr J. Millo, together with other members of the GenOSept Consortium. Variables recorded pertained to demographic, clinical and outcome data. A specific electronic case report form (eCRF) was developed by Lincoln, Paris, France, using software developed in collaboration with JDC. The database was password protected, allowing investigators to enter data into the eCRF online, and included audit trail capability for data entry and subsequent modifications. To minimize errors logical range checks were in place so that the investigators would be alerted if an attempt was made to enter data values outside the expected ranges.

Quality Assurance (QA) was performed by P.H., C.G., A.W., A.G. and C.H, who systematically reviewed all data. Data queries (DQs) were generated within the eCRF for missing or erroneous data, and sent electronically to the relevant investigators for action, where necessary. Up to the end of January 2011 an estimated 3986 valid DQs had been generated, with a response rate by the investigators of approximately 92%. Common reasons for DQs were missing information, particularly the Charleson Index, antimicrobial use, estimated day of onset of FP before ICU admission, information about circumstances of GCS assessment, and outcome data.

All patients’ eCRFs were reviewed by experienced critical care physicians. Where the patient’s eligibility for inclusion in the relevant cohort was unclear, clarification was sought from the investigators. Regular QA reports were provided to the GenOSept Management Committee for review; the National Investigators were contacted regarding quality issues if necessary. Of the original 1123 records pertaining to patients with any type of peritonitis, 146 were eliminated following QA, as not meeting the criteria for definition of peritonitis of faecal origin, leaving 977 patient records with FP.

**List of ethical bodies that approved the study**

REC, Research Ethics Committee

| Ethic Commissions/Bodies |  |
| --- | --- |
| Ethikkommission  Land Salzburg Ethik Kommission fur das Bundesland Salzburg | Ethikkommission für das Bundesland Salzburg Sebastian-Stief-Gasse 2 5020 Salzburg  Postanschrift:  Amt der Salzburger Landesregierung Ethikkommission für das Bundesland Salzburg Postfach 527 5010 Salzburg |
| Comite d’Ethique ISPPC | Chu - Charleroi  Boulevard Zoé Drion, 1  6000 Charleroi |
| Comite d’Ethique Hospitalo-Faculatiare de Liege | Centre Hospitalier Universitaire de Liège  Domaine Universitaire du Sart Tilman  Bâtiment B 35  B-4000 Liège  Belgique  Clinique Saint Pierre, Avenue Reine Faibiola, 9  1340 Ottignies |
| Multicenter Ethics Committee  Stanovisko multicentricke Eticke Komis Fakultni Nemoncnice U SV. Anny V Brno | Faculty hospital Brno  Jihlavská 20  625 00 Brno  Czech Republic |
| Ethics Committee of Faculty Hospital Hradec Kralove | Sokolaska 581, 500 05 Hradec  Czech Republic |
| Ethics Committee of the University Hospital and Faculty of Medicine Palacky University in Olomouc | I.P. Pavlova 6, 775 20 Olomouc  Czech Republic |
| Ethics Committee at University Hospital Ostrava | Ethics Committee  Fakultní nemocnice s poliklinikou Ostrava  17. listopadu 1790, 708 52 Ostrava  Poruba,  Czech Republic |
| Ethics Committee  Rozhodnuti Eticke Komise Fakultni Nemocnice Pizen | FN a LF UK Plzeň  tř. Dr. E. Beneše 13  305 99 Plzeň  Czech Republic |
| Ethics Committee of Masaryk’s Hospital Usti n.Labem | Socialni pece 3316/12 A  401 13 Usti n. Labem  Czech Republic |
| Eticka Komise Nemocnice Znojmo | Nemocnice Znojmo, příspěvková organizace  MUDr. Jana Janského 11 669 02 Znojmo  Czech Republic |
| Ethics Committee for multicenter clinical trials County Hospital Liberec | Husova 10, Liberec,  Czech Republic |
| Eticka Komise pro multicentricka hodnoceni  Krajska nemocnice Liberec | Liberec, Husova 10  460 63 Liberec 1  Czech Republic |
| Ethics Committee on Human Research of the University of Tartu | TU Biomeedikum  Room 3050  Ravila Str 19  51014, Tartu,  Estonia |
| Ethik Kommission Landesarztekammer  Rheinland-Pfalz | Postfach 29 26 55019 Mainz  Germany |
| Ethik Kommission Arztekammer Mecklenburg-Vorpommern | Ernst Moritz Amdt Universitat Greifswald, Friedrich-Loeffler Str. 23d 17487 Greifswald  Germany |
| Ethik Kommission Bayerishe Landesarztekammer | Mühlbaurstr.16 D-81677 München  Germany |
| Ethik commission Technische Universtat Dresden | Ethikkommission Technische Universität Dresden Fetscherstraße 74 01307 Dresden Germany |
| Ethik Kommission Univesitatsklinikum  Rheinisch Westfalische Technische Hochschule Aachen | Universitätsklinikum Aachen Pauwelsstraße 30 52074 Aachen  Germany |
| Ethik Kommission Universitatslinikum Jena der Friedrich –Schiller | Bachstrasse 18, D-07740 Jena  Germany |
| Ethik Kommission  Schleswig-Holstein | Arnold-Heller-Straße 3 - Haus 18 24105 Kiel  Germany |
| Ethik Kommission for Arztekammer Hamburg | Humboldstrasse 67a – 22083 Hamburg  Germany |
| Ethik Kommission  Universitat Heidelberg | Maybackstrasse 14-15, D68169 Mannheim  Germany |
| Ethik Kommission  Friedrich-alexander Universitat Erlangen-Nuremberg | Ethik-Kommission der Medizinischen Fakultät der Friedrich-Alexander-Universität Erlangen-Nürnberg  Krankenhausstraße 12 91054 Erlangen  Germany |
| Ethik Kommission  Rheinische Friedrich-Wilhelms Universitat | Reuterstr. 2b  53113 Bonn  Germany |
| Ethik Kommission  Universitat Ulm | Helmholtzstrasse 20  D-89081 Ulm  Germany |
| Ethik Kommission Arztekammer des Saarlandes | Faktorestrasse 4  66111 Saarbrucken  Germany |
| Helsinki Ethics Committee | Kiryat Hadassah,  POB 12000  Jerusalem, 91120,  Israel |
| Sismanoglio Geniko Nosokomeio | Sismanogliou 1  Marousi 151 26  Greece |
| Ethics Commission University College Cork, Ireland | Lancaster Hall, 6 Little Hanover Street, Cork, Ireland |
| Ethics Commission The Adelaide & Meath Hospital, Dublin | Tallaght, Dublin 24,  Ireland |
| Ethics Commission Merlin Park Hospital, Galway | Unit 4,  Merlin Park Hospital  Galway  Ireland |
| Comitato Etico dell’Azienda Sanitaria Ospedaliera “San Giovanni Battista” di Torino | Corso Bramante  88-90  10126  Torino  Italy |
| Comitato Etico  Universita’ degli studi di Napoli Federico II  comitato etico per le attivita’ biomediche | Via Sergio Pansini 5  80131 Napoli  Italy |
| Comitato Etico  Azienda Ospedaliiero Universitaria Ospedali Rhuniti, Ancona | VIA CONCA 71 60126  ANCONA (Ancona)  Italy |
| Comitato Etico  Azienda Ospedaliera Universitaria Careggi, Firenze | Viale Peiraccini 28  50139 Firenze  Italy |
| Comitato Etico  Azienda Ospedaliera San Gerardo, Monza | Via Pergolesi 33  20053 Monza (MI)  Italy |
| Comitato Etico  Azienda Ospedaliero Universitaria Di Ferrara | Via A.moro 8  Cona (FE)  Italy |
| Ethische Commissie Erasmus MC Universitai Medisch Centrum Rotterdam | Postbus 2040  3000 Ca  Rotterdam  Netherlands |
| Komisja Karol Marcinkowski University of Medical Sciences in Poznan | Collegium Maius  Fredry 10  61-701 Poznań  Poland |
| Ethics Committee of Military Medical Academy | 17 Crnotravska  Serbia |
| Ethics Committee of Clinical Center Kragujevac | CLINICAL CENTER KRAGUJEVAC  Zmaj Jovina street 30  Kragujevac  Serbia |
| Comite Etico de Investigacion Clinica del Hospital General Universitario de Alicante | Hospital General Universitario de Alicante  Pintor Baeza, 12,  03010 Alicante,  Spain |
| Comite Etico Hospital Universitario Dr. Peset | Av. de Gaspar Aguilar, 90,  46017 València,  Valencia,  Spain |
| Comite Etico de Investigacion Clinica del Hospital de Bellvitge Barcelona | Feixa Llarga, s/n, 08907  L'Hospitalet de Llobregat,  Barcelona,  Spain |
| Comite Etico  Hospital Universitario Puerta Del Mar | Av. Ana de Viya, 21,  11009 Cádiz,  Spain |
| Comite Etico  Hospital Universitario de Gran Canaria | Calle Dr. Alfonso Chiscano Díaz, 338  35010 Las Palmas de Gran Canaria,  Las Palmas,  Spain |
| Comite Etico  Hospital Universitario de La Princesa Madrid | Calle de Diego Leon, 62,  28006 Madrid,  Spain |
| Comite Etico  Hospital General Universitario Reina Sofia de Murcia | Av. Intendente Jorge Palacios, 1,  Murcia,  Spain |
| Comite Etico  Hospital Virgen de la Victoria Malaga | Campus de Teatinos, s/n,  29010 Málaga,  Spain |
| Comite Etico  Hospital De Mataro, Consorci Sanitari del Maresme | Carrer Prolongació Cirera, s/n,  08304 Mataró,  Barcelona,  Spain |
| Comite Etico  Hospital Clinico San Carlos | Profesor Martín Lagos, S/N  Madrid - 28040  Spain |
| Comite Etico  Hospital Universitari De Tarragona Joan XXIII | C/ Dr. Mallafrè Guasch, 4,  43005 Tarragona,  Spain |
| Comite Etico  Hospital de Sagunto | Avenida Ramón y Cajal, S/N,  46520 Sagunt, Valencia,  Spain |
| Comite Etico  Centro Medico Delfos | Av. de Vallcarca, 151,  08023 Barcelona,  Spain |
| Comite Etico  Hospital Universitari Joan XXIII de Tarragona | C/ Dr. Mallafrè Guasch, 4,  43005 Tarragona,  Spain |
| Comite Etico de investigacion  Clinica De Aragon (CEICA) | Avenida Gomez Laguna  25 planta 3  50009 Zaragoza  Spain |
| Comite Etico  Hospital de Basurto | Montevideo Etorb., 18,  48013 Bilbao, Vizcaya, Bizkaia,  Spain |
| Comite Etico  Hospital Santa Maria del Rosell | Paseo de Alfonso XIII, 61 ,  30203 Cartagena ,  Spain |
| Comite Etico  Hospital Universitario Arnau de Vilanova de Lleida | Avenida Alcalde Rovira Roure, 80  25198 Lleida  Spain |
| Comite Etico  Hospital Universitario de Burgos | Avda. Islas Baleares, 3,  09006 Burgos,  Spain |
| Comite Etico  Hospital Universitario Puerto Real | Carretera Nacional IV, km 665, 11510  Puerto Real, Cádiz,  Spain |
| Comite Etico  Hospital Universitari de Girona Doctor Josep Trueta | Avenida França, s/n,  17007 Girona,  Spain |
| Comite D’Etica d’Investigacio Clinica  Consorci Hospitalari de Vic | Av. de França, s/n 9a planta A -  Despatx 913  17007 - Girona  Spain |
| BARTS AND THE LONDON NHS TRUST | East London and the City HA Local REC 2 |
| JOHN RADCLIFFE HOSPITAL | Oxfordshire REC B |
| Addenbrooke's Hospital | Cambridhe REC |
| Newcastle upon Tyne Hospitals NHS Trust | Newcastle and North Tyneside Local REC 2 |
| University Hospital of Leicester NHS Trust | Leicestershire, Northamptonshire and Rutland REC 1 |
| THE GREAT WESTERN HOSPITAL, Swindon | Swindon REC |
| WORTHING HOSPITAL | West Sussex Local REC |
| QUEEN ELIZABETH UNIVERSITY HOSPITAL | South Birmingham REC |
| SOUTHEND HOSPITAL NHS TRUST | South-Essex REC |
| Lewisham Hospital NHS Trust | Lewisham Hospital Local REC |
| IPSWICH HOSPITAL NHS TRUST | Suffolk Local REC |
| FRIMLEY PARK HOSPITAL | South West Surrey Local REC |
| Royal Berkshire Hospital | Berkshire REC |
| UCLH Hospitals  NHS Trust | The National Hospital for Neurology and Neurosurgery and Institute of Neurology joint REC |
| NORFOLK & NORWICH NHS TRUST | Norfolk REC |
| Wythenshawe Hospital | South Manchester Local REC |
| The Leeds Teaching Hospitals | Leeds (East) and Leeds (West) RECs |
| CHELTENHAM GENERAL HOSPITAL | Gloucestershire REC |
| Kings College Hospital | Kings College Hospital REC |
| THE JAMES COOK UNIVERSITY HOSPITAL | South Tees Local REC |
| Queen Elizabeth Hospital, King's Lynn | Norfolk REC |
| Sheffield Teaching Hospitals NHS Trust / Royal Hallamshire Hospital | South Sheffield REC |
| NHS GRampian | Grampian Local REC |
| Mid Essex Hospital Services NHS Trust | North and Mid Essex Local REC |
| Brighton and Sussex University Hospitals NHS Trust | Brighton East REC |

**List of Contributing Centres and Investigators**

*Belgium*:

Intensive Care Unit, AZ-VUB university hospital, 101 Laarbeeklaan, Brussels; Intensive Care Unit, Chu Charleroi, 92 Boulevard Janson, Charleroi; Soins Intensifs, Clinique Saint Pierre, 9 Avenue Reine Fabiola, Ottignies; Intensive Care, Cliniques Universitaires Saint Luc (UCL), 10 Avenue Hippocrate, Brussels; Intensive Care, University hospital, 185 De Pintelaan, Gent; Soins Intensifs, Cliniques de l'europe - St Michel,150 Rue de Linthout, Brussels.

*Croatia*:

Medic, Emergency and Intensive Care Medicine/Internal Medicine, Clinical hospital Rebro, 12 Kispaticeva, ZagrebAnestesiology and ICU, Clinical Hospital Rebro, 12 Kispaticeva, Zagreb.

*Czech Republic*:

Anesteziologicko-reuscitacni klinika, Fakultní Nemocnice u Svaté Anny, 53 Pekařská, Brno; Anesteziologicko-resuscitacni oddeleni, Fakultni Nemocnice Brno, 20 Jihlavská, Brno-Bohunice; Klinika anestezie, resuscitace a intenzivni mediciny, Fakultni Nemocnice Hradec Kralove, 581 Sokolská, Hradec Kralove; Chirurgicka klinika, Fakultní Nemocnice s Poliklinikou Ostrava, 1790 listopadu, Ostrava-Poruba; Anesteziologicko-resuscitacni klinika, Fakultni Nemocnice Plzen, 80 Alej Svobody Plzen; Anestezie, resuscitace a intenzivni medicina, Masarykova Nemocnice, 3316/12A Sociální péče, Ústi Nad Labem; Anesteziologicko-resuscitacni oddeleni, Nemocnice Znojmo, 11 Janského, Znojmo; Anesteziologicko-resuscitacni oddeleni, Krajska Nemocnice Liberec, 10 Husova, Liberec.

*Estonia*:

General ICU, Tartu University Hospital, 1a L. Puusepa, Tartu; Pulmonary ICU, Tartu University Hospital,1a L. Puusepa, Tartu.

*Franc*e:

Service de Réanimation Médicale, Hopital Cochin, 27 rue du Fbg St Jacques, Paris; Service de Réanimation Médicale, HEGP, 20 rue Leblanc, Paris; Service de Réanimation Médicale, Hotel Dieu, 1 place du Parvis Notre Dame, Paris; Service de Réanimation Médicale, Saint Joseph, 185 rue Raymond Losserand, Paris; Service de Réanimation Médicale, Chru Angers, 4 rue Larrey, Angers; Service de Réanimation Médicale, Chu de Nice, Rte St Antoine Ginestière, Nice; Service de Réanimation Médicale, Chu Purpan, Chu Toulouse- Hôpital Purpan, Toulouse; Service de Réanimation Médicale, Ch Versailles, 177 rue de Versailles, Le Chesnay.

*Germany*:

Klinik für Herzchirurgie, Klinikum der Stadt Ludwigshafen am Rhein GGMBH, 79 Bremserstraße, Ludwigshafen; Klinik und Poliklinik für Anästhesiologie und Intensivmedizin, Klinikum Greifswald, 23b Friedrich-Loeffler-Straße, Greifswald; Klinik fur Anästhesiologie und operative Intensivmedizin, Klinikum Augsburg, 2 Stenglinstr., Augsburg; Klinik und Poliklinik für Anaesthesiologie und Intensivtherapie, Universitätsklinikum Dresden, 74 Fetscherstrasse, Dresden; Klinik für Anästhesiologie und Intensivtherapie, Klinikum der Friedrich Schiller Universität, 101 Erlanger Allee, Jena; Klinik für Anästhesie und Intensivmedizin, Westküstenklinikum Heide, 50 Esmarchstraße, Heide; Abt. fur Anästhesiologie und Intensivtherapie, Fachkrankenhaus Coswig - centre for pneumology and thoracic surgery, 21 Neucoswiger Str., Coswig; Klinikum der Medizinischen Fakultät der Martin Luther Universität Halle-Wittenberg, 40 Ernst-Grube-Str., Halle; Klinik für Intensivmedizin, University medical center Eppendorf, 52 Martinistr., Hamburg; Klinik und Poliklinik für Anästhesiologie und Operative Intensivmedizin (Turmgebäude 2OG Zimmer 221), Universitätsklinikum Bonn, 25 Sigmund-Freud-Str., Bonn; Internal Medicine, Universitätsklinikum Mainz, 1 Langenbeckstrasse, Mainz.

*Greece*:

Intensive Care, Sismanoglion general hospital, Marousi, Athens; Critical care, Attikon university hospital, 1 Rimini, Xaidari.

*Hungary*:

Surgery 1^St^, Semmelweis University, 78 Ulloi Ut, Budapest

*Eire*:

Intensive care unit, St James hospital, James Street, Dublin; Intensive care unit, Adelaide Meath and national children’s hospital, Tallaght, Dublin; Anaesthesia and Intensive care, National university hospital Galway, Newcastle Road, Galway; Anaesthesia & Intensive Care Medicine, James Connolly memorial hospital, Blanchardstown, Dublin; Department of Anaesthesia and Intensive Care Medicine, Cork university hospital, Wilton, Cork.

*Israel*:

Carmel medical center, Haifa; General Intensive care unit, Haemek medical center, Afula; Anaesthesiology and critical care medicine, Hadassah medical center, Kiryat Hadassah, P.O. Box 12000, Jerusalem

*Italy*:

Anestesiologia e Rianimazione 3, Ospedale S. Giovanni Battista – Molinette, 88 Corso Bramante, Torino; Anestesia e Rianimazione, Ospedale S.Giovanni Bosco, 3 Piazza Donatori del Sangue, Torino; Dr Rianimazione SOD 2, AOU Careggi, 85 Viale Morgagni, Firenze; Anestesia e Rianimazione, Ospedale Maggiore, 35 Via Francesco Sforza, Milano; Terapia Intensiva, Universita Degli Studi Milano Bicocca A.O. San Gerardo, 106 Via Donizetti, Monza; Anestesia e Rianimazione, Ospedale S.Orsola Malpighi, 9 Via Massarenti, Bologna; Anestesia e Rianimazione, Ospedale S.Giovanni Addolorata, 8 Via dell'Amba Aradam, Roma; Scienze Anestesiologische, Medicina Critica e Terapia del Dolore, Policlinico Umberto I, 155 Viale del Policlinico, Roma.

*Netherlands*:

Intensive care unit, Erasmus medical centre, 230 Gravendijkwal, Rotterdam.

*Poland*:

Anaesthesiology and Intensive Therapy, Medical university, 7 Debinki St, Gdansk; Klinika Anestezjologii i Intensywnej Terapii sp Centralny Szpital Kliniczny Sam; Military teaching hospital; Szpital Wojewodzki/regional hospital; University hospital n°2; Szpital Wojewodzki; University hospital of Bydgoszcz; Wroclaw medical University.

*Serbia*:

Military medical academy; Clinical center Kragujevac.

*Spain*:

Coordinating centre: Universitat Roira & Virgili / Hospital Universitari Joan XXIII de Tarragona, CIBERES. University hospital de Bellvitge; hospital Universitario Puerta del Mar; hospital Universitario de Gran Canaria; hospital de la Princesa; hospital Nostra Senyora de Meritxell; hospital de Mataro; hospital clinico San Carlos; hospital Universitari de Terragona Joan XXIII; hospital Sagunt; centro medico Delfos; hospital de Huesca; hospital general de Segovia; Basurto hospital; hospital Universitario Arnau de Vilanova; hospital general Yague; hospital Universitario Puerto Real; hospital Universitario de Girona; hospital General de Vic; Hospital Verge De La Cinta.

*United Kingdom*:

Aberdeen Royal Infirmary; Addenbrooke's Hospital; Barts and the London NHS trust; Broomfield hospital; Charing Cross Hospital; Chelsea and Westminster Hospital; Cheltenham general hospital; Colchester General Hospital; Freeman Hospital; Frimley Park hospital; Hammersmith hospital; Homerton University hospital; Hope hospital; Huddersfield royal infirmary; Hull royal infirmary; Ipswich hospital NHS trust; John Radcliffe hospital; Leeds general infirmary; Leicester royal infirmary; Manor hospital, Walsall; Norfolk & Norwich NHS trust; Queen Elizabeth hospital, King's Lynn ; Queen Elizabeth University hospital; Royal Berkshire Hospital; Royal Hallamshire hospital, Sheffield; Royal Preston hospital; Royal Sussex county hospital; Royal Victoria infirmary; Southend hospital NHS trust; st James University hospital; the Great Western hospital; the James Cook University hospital; The Whittington hospital; UCLH Middlesex hospital; University hospital Lewisham; University hospital of Wales; University hospital, Coventry; Worthing hospital; Wythenshawe Hospital

**National Co-ordinators:**

| Austria | H Novak |
| --- | --- |
| Belgium | P Damas |
| Croatia | V Gasparovic |
| Czech Republic | V Sramek |
| Estonia | S Sarapuu |
| France | J-D Chiche |
| Germany | F Bloos |
| Greece | A Armagandis |
| Hungary | I Bobek |
| Ireland | T Ryan |
| Israel | Y Weiss |
| Italy | P Cotogni |
| Netherlands | J Hazelzet |
| Poland | A Mikstacki |
| Serbia | M Surbatovic |
| Spain | J Rello |
| United Kingdom | C Hinds |

**Principal Investigators**

**Austria AT**

H Novak

Belgium BE

| H Spapen  P Biston  T Dugernier  P.F. Laterre  P Damas  V Collin |  |
| --- | --- |
| **Croatia HR**  M Grgic Medic  T Mahecic |  |
| **Czech Republic CZ**  V Sramek  J Mannova  D Bares  O Marek  I Satinsky  I Novak  M Panko  S Vojtech  I Zykova |  |
| **Estonia EE**  S Sarapuu  **France FR**  J D Chiche  J L Diehl  A Rabbat  B Misset  P Asfar  H Hyvernat  P Sanchez  J-P Bedos  **Germany DE**  F Isgro  M Grundling  U Jaschinski  M Ragaller  F Bloos  S Schroder  J Krassler  A Nierhaus  C Putensen  M Weiss  Prof Larsen  M Lauterbach  **Greece GR**  D Evrenoglou  A Armaganidis  **Hungary HU**  K Darvas  I Okros  **Ireland IE**  T Ryan  M Donnelly  J Laffey  C Cody  C Motherway  D Breen  **Israel IL**  R Pizov  A Lev  Y Weiss  **Italy IT**  V. M Ranieri  S Livigni  P Pelaia  R Tufano  A.R De Gaudio  L Gattinoni  A Pesenti  M Capuzzo  G Sangiorgi  F Turani  F Conforto  F Bilotta |  |

**Netherlands NL**

B Van Der Hoven

Poland PL

| A Siemiatkowski |
| --- |
| D Maciejewski |
| M Wujtewicz |
| E Karpel |
| A Ziajka |
| R Gajdosz |
| W Gaszynski |
| A Nestorowic |
| W Kowalski |
| A Mikstacki |
| L Drobnik |
| L Krawczyk |
| J Jastrzebski |
| A Kanski |
| W Koscielniak |
| M Mikaszweska-Sokolewicz |
| K Kusza |
| A Kubler |
| B Jozef |

Serbia RS

| M Surbatovic |
| --- |
| J Jevdjic |

Spain ES

| X L Perez-Fernandez |
| --- |
| R l Sierra |
| J Sole-Violan |
| N Carrasco |
| A Margarit-Ribas |
| J C Yebenes |
| A Valverde-Conde |
| G Sirgo |
| E Gomez-Martinez |
| F F Dorado |
| L Labarta |
| L Cambra |
| M A Vidarte-Ortiz |
| M B Castello |
| J L Fernandez |
| J Gil Cebrian |
| J M Sirvent |
| M C Martin |

United Kingdom UK

| C Hinds  C Garrard  A Johnston  D Watson  S Baudouin  M Watters  R Venn  J Bion  D Higgins  M J. Garfield  S Pambakian  J Thompson  J Durcan  A Kapila  G Bellingan  S Fletcher  A Bentley  A Mallick  R Bailie  I Krupe  M Oram  M Hayes  E Wheatley  S Murdoch  S Bonner  N Webster  G Findlay  M Blunt  G Mills  G Thomas  S Drage  A Timmins  S Pesian  A Gordon  M Kuper  P Hall  P Venkatesh  J Moreno Cuesta  S Laha  A Guleri  I Smith  A Krige  P Watt |
| --- |
| **Research Nurses/Fellows** |
| E Svoren  A Purdy  E McLees  P Hutton  P Parsons  A Smith  R Farras-Arraya  C Higham  C Ryan  C Pirie  K Mayell  K Challis  S Morris  N Waterhouse  V Flitchett  J Margalef  Dr Mowatt  P Hudson  R Gupta  J Wilde  S Lees  A Nillson  S Andrews  E Simpson  S Mappleback  S Burfield  L Sherrard Smith  V Jamieson  K Williamson  E Thomson  S Rogers  N Wilson  S Bowrey  N Rich  N Griffin-Teal  C Mitchell-Inwang  S Williams  K Swan  S Smolen  C Jones  H Prowse  N Jacques  J Atkinson  S Boluda  A Bakarr Karim  J Hyun Ryu  J Nagle  G Bercades  M Rosbergen  G Glister  F Jefferies  D Downs  K Millward  S Elliot  J Thornton  D Mawer  J Calderwood  I Whitehead  V Goodridge  K Hugill  K Colling  S Roughton  H Tennant  J Taylor  S Hall  J Addison  L Macchiovello  E Hutcheon  C Underwood  K Wong  J Collins  N Mills  E Calton  J Sorrell  S Lowes  L Ortiz-Ruiz De Gordoa  A Ghosh  O Thunder  N Wheatley  M Templeton  R Wilson  C Gibbs  L Mountford  J Gonzalez-Moreno  M Ainsworth  S Pahary  S Musaad  J Hewlett  J England  G Ward  S Nyabadza  S Clay  C Gibson  E Archer  K Hotchkiss  D Gocher  J Daglish  M Dlamini  J Baldwin  N Doherty  J Cocker  N Waddington  N Smith  D Harrison  M Bland  L Bullock  P Raymode  G Sirgo  T Lisboa  E Diaz |
